# Mitochondrial proteomics with siRNA knockdown to reveal ACAT1 and MDH2 in the development of doxorubicin-resistant uterine cancer

**DOI:** 10.1111/jcmm.12388

**Published:** 2015-01-30

**Authors:** Yi-Wen Lo, Szu-Ting Lin, Shing-Jyh Chang, Chia-Hao Chan, Kevin W Lyu, Jo-Fan Chang, Eugenie Wong Soon May, Dai-Ying Lin, Hsiu-Chuan Chou, Hong-Lin Chan

**Affiliations:** a Department of Applied Science, National Hsinchu University of EducationHsinchu, Taiwan; b Institute of Bioinformatics and Structural Biology and Department of Medical Sciences, National Tsing Hua UniversityHsinchu, Taiwan; c Gynecologic Oncology Section Department of Obstetrics and Gynecology, Hsinchu Mackay Memorial HospitalHsinchu, Taiwan; d Lutheran Medical CenterBrooklyn, NY, USA; e Global Scholars Program, St. George's University/Northumbria UniversityNewcastle upon Tyne, UK

**Keywords:** mitochondrial, DIGE, doxorubicin, resistance, uterine cancer

## Abstract

Mitochondria are key organelles in mammary cells in responsible for a number of cellular functions including cell survival and energy metabolism. Moreover, mitochondria are one of the major targets under doxorubicin treatment. In this study, low-abundant mitochondrial proteins were enriched for proteomic analysis with the state-of-the-art two-dimensional differential gel electrophoresis (2D-DIGE) and matrix-assistant laser desorption ionization time-of-flight mass spectrometry (MALDI-TOF MS) strategy to compare and identify the mitochondrial protein profiling changes in response to the development of doxorubicin resistance in human uterine cancer cells. The mitochondrial proteomic results demonstrate more than fifteen hundred protein features were resolved from the equal amount pooled of three purified mitochondrial proteins and 101 differentially expressed spots were identified. In which, 39 out of these 101 identified proteins belong to mitochondrial proteins. Mitochondrial proteins such as acetyl-CoA acetyltransferase (ACAT1) and malate dehydrogenase (MDH2) have not been reported with the roles on the formation of doxorubicin resistance in our knowledge. Further studies have used RNA interference and cell viability analysis to evidence the essential roles of ACAT1 and MDH2 on their potency in the formation of doxorubicin resistance through increased cell viability and decreased cell apoptosis during doxorubicin treatment. To sum up, our current mitochondrial proteomic approaches allowed us to identify numerous proteins, including ACAT1 and MDH2, involved in various drug-resistance-forming mechanisms. Our results provide potential diagnostic markers and therapeutic candidates for the treatment of doxorubicin-resistant uterine cancer.

## Introduction

Doxorubicin is one of primary anticancer drugs used in the treatment of a broad range of tumour types 1–3. However, doxorubicin's precise anti-cancer mechanisms are complex; previous study showed that doxorubicin disturbs DNA replication by intercalation into DNA double strand and leads to the subsequent inhibition of DNA, RNA and proteins synthesis. Moreover, doxorubicin has been reported to inhibit topoisomerase 2 and leads to the breakage of DNA double strand which prevents DNA synthesis. In addition, doxorubicin directly damages cancer cells through disturbing the normal physiology of mitochondria resulting in cell apoptosis 4,5. Nevertheless, doxorubicin-induced chemotherapy resistance has been widely reported in cancer chemotherapy such as breast cancer, lung cancer, leukaemia, osteosarcoma and uterine cancer 6–10, and is a primary therapeutic problem to the successful treatment of patients who receive cancer chemotherapy.

Chemotherapy resistance diminishes the anti-cancer effects of chemotherapy drugs used in the treatment of tumours. In clinical practice, chemotherapy resistance has been recognized as a severe problem when the concentrations of chemotherapy drugs approach at harmful and toxic dosages to kill tumours. Biological mechanisms associated with drug resistance have been described including the increased activities of plasma membrane drug efflux transporter such as adenosine triphosphate binding cassette-transporters, the enhanced detoxification enzymes such as glutathione reductase and the activation of cellular signalling pathways that direct cell survival and cell death 11. However, not all of chemotherapy resistance can be completely explained by the elucidated mechanisms. Consequently, it is important to globally investigate the additional resistance mechanisms which have not yet to be clarified.

Because the development of chemotherapy resistance is multiple factors and related to the alterations in numerous proteins, it is advantageous to use proteomic strategies to globally determine chemotherapy resistance-related proteins. Our previous proteomic analysis has identified numerous proteins, including asparagine synthetase and membrane-associated progesterone receptor component 1, involved in a variety of drug-resistance-forming mechanisms 12. In this study, to decrease complexity and discover low abundance proteins, subcellular proteomic analysis based on purification of mitochondrial proteins were performed to study the correlation between mitochondria and doxorubicin-induced chemotherapy resistance since mitochondria are crucial for the survival of resistance cancer cells and their defects contribute to the development of chemotherapy resistance 13,14. To carry out an *in vitro* investigation into doxorubicin-resistance mechanisms in uterine cancer, increase our understanding of the molecular mechanisms involved, and identify potential chemotherapy resistance biomarkers with possible diagnostic or therapeutic applications, we established a serial of uterine sarcoma cancer lines, MES-SA, and its doxorubicin-resistant partners MES-SA/Dx-2 μM cells and MES-SA/Dx-8 μM cells as a model system to examine chemotherapy resistance-dependent mitochondrial protein alterations *via* quantitative proteomic analysis with 2D-DIGE and MALDI-TOF mass spectrometry. This study also includes reports of studies that used siRNA silencing against selected identified proteins, ACAT1 and MDH2, to monitor and evaluate their potency against doxorubicin resistance.

## Materials and methods

### Chemical and reagents

Generic chemicals were purchased from Sigma-Aldrich (St. Louis, MO, USA), while reagents for 2D-DIGE were purchased from GE Healthcare (Uppsala, Sweden). All primary antibodies were purchased from Genetex (Hsinchu, Taiwan) and antimouse and anti-rabbit secondary antibodies were purchased from GE Healthcare. All the chemicals and biochemicals used in this study were of analytical grade.

### Cell lines and cell cultures

The uterine sarcoma cancer line MES-SA was purchased from American Type Culture Collection (Manassas, VA, USA) and cultured in McCoy's 5a modified medium containing 10% foetal bovine serum, L-glutamine (2 mM), streptomycin (100 μg/ml), penicillin (100 IU/ml) (all from Gibco-Invitrogen Corp., Paisley, UK). The doxorubicin-resistance lines MES-SA/Dx-2 μM and MES-SA/Dx-8 μM cell were both derived from MES-SA *via* stepwise increasing the doxorubicin concentrations in medium, and were maintained in the same medium and supplement with 0.2 μM and 0.8 μM doxorubicin respectively. All cells were incubated at 37°C in a humidified atmosphere containing 5% CO_2_. The IC_50_ values for MES-SA and its doxorubicin-resistance lines MES-SA/Dx-2 μM and MES-SA/Dx-8 μM were 0.25 μM, 5.31 μM and 18.75 μM respectively.

### Sample preparation for mitochondrial proteomic analysis

Mitochondria were isolated by using the mitochondrial isolation kit for mammalian cells (Millipore, Darmstadt, Germany) according to our previous report 15. Briefly, following lysis of ∼2.5 × 10^7^ of MES-SA, MES-SA/Dx-2 μM or MES-SA/Dx-8 μM, cell debris and nuclei were pelleted at 700 × g, followed by centrifugation at 5000 × g to pellet a mitochondrially enriched fraction. The crude mitochondria were washed in chilled 0.5× PBS and lysed in 2-DE lysis buffer containing 4% w/v CHAPS, 7 M urea, 2 M thiourea, 10 mM Tris-HCl, pH 8.3, 1 mM EDTA. Lysates were homogenized by passage through a 25-gauge needle for 10 times and the insoluble material removed by centrifugation at 13,000 r.p.m. for 30 min. at 4°C and protein concentrations were determined by using the coomassie protein assay reagent (Bio-Rad, Hercules, CA, USA).

### MTT cell viability assay

The detailed MTT experimental procedure has been described in our previous study 12.

### Mitochondrial membrane potential assay by JC-10 fluorescence and flow cytometry

The mitochondrial membrane potentials of cultured cells were determined by using the fluorescent probe JC-10 (AAT Bioquest, Sunnyvale, CA, USA) following the manufacturer's recommendations. Briefly, cultured cells were exposed to 5 μM mitochondrial uncoupler carbonyl cyanide-4-(trifluoromethoxy) phenylhydrazone (FCCP) for 1 hr and then incubated in culture medium containing JC-10 for 1 hr at room temperature. The cells were washed with PBS and analysed by flow cytometry. Photomultiplier settings were adjusted to detect JC-10 monomer and aggregate fluorescence on the FL1 (525 nm) and FL2 (595 nm) detectors. The fluorescence ratio at these wavelengths was used to monitor changes in mitochondrial membrane potential.

### Flow cytometry analysis for apoptosis detection

Annexin V/propidium iodide (PI) double assay was performed with the Annexin V, Alexa Fluor® 488 Conjugate Detection kit (Life Technologies, Paisley, UK). Briefly, 10^6^ cells were resuspended in 500 μl binding buffer and stained with 5 μl Alexa Fluor 488 conjugated annexin V according to the manufacturer's instructions. 1 μl 100 μg/ml PI was added and mixed gently to incubate with cells for 15 min. at room temperature in the dark. After incubation period, samples were subjected to FCM analysis in 1 hr. by using BD Accuri C6 Flow Cytometry (BD Biosciences, San Jose, CA, USA). The data were analysed by using Accuri CFlow^@^ and CFlow Plus analysis software (BD Biosciences).

### 2D-DIGE, gel image analysis, protein staining, in-gel digestion and MALDI-TOF MS analysis

The detailed experimental procedures have been described in our previous publications 16–18. Notably, peaks in the mass range of *m/z* 800-3000 were used to generate a peptide mass fingerprint that was searched against the Swiss-Prot/TrEMBL database (released on November 2011) with 533,049 entries by using Mascot software v2.3.02 (Matrix Science, London, UK). The parameters used for Mascot search are listed: *Homo sapiens*; tryptic digest with a maximum of 1 missed cleavage; carbamidomethylation of cysteine, partial protein N-terminal acetylation, partial methionine oxidation and partial modification of glutamine to pyroglutamate and a mass tolerance of 50 ppm. Identification was accepted based on significant MASCOT Mowse scores (*P* < 0.05), spectrum annotation and observed *versus* expected molecular weight and p*I* on 2-DE as well as at least 5 peptides in each identified protein.

### Immunoblotting analysis

Immunoblotting analysis was used to validate the differential abundance of mass spectrometry identified proteins across MES-SA, MES-SA/Dx-2 μM and MES-SA/Dx-8 μM cells. The detailed experimental protocol has been described in our previous study 19. All primary antibodies used for expression validation were purchased from Genetex.

### siRNA design, construction and transfection

The siRNA against ACAT1 and MDH2 were synthesized by Invitrogen. The targeting sequences 5′-UAU UGU AGA CAU CAG UUA GCC CGU C-3′ against ACAT1, and 5′-UAA CGA AGG AAC AUU CCA CAA CAC C-3′ against MDH2 were designed and verified to be specific by Blast search against the human genome, and sequences of similar GC contents which do not match any known human coding sequence were used for negative control against ACAT1 and MDH2. Transfection was mediated with Lipofectamine RNAiMAX (Invitrogen) according to the manufacturer's instruction. Briefly, cells were transfected with 20 nM of ACAT1 siRNA and 20 nM of MDH2 siRNA or the corresponding control (pGCsi-control) in serum free medium containing Lipofectamine RNAiMAX for 4 hrs followed by recovered in medium containing 10% FCS for 24 hrs. The efficiency of siRNA knockdown was monitored with immunoblotting by using primary antibodies against ACAT1 and MDH2.

## Results

### Development of doxorubicin-resistance uterine cancer lines

**Fig 1:**
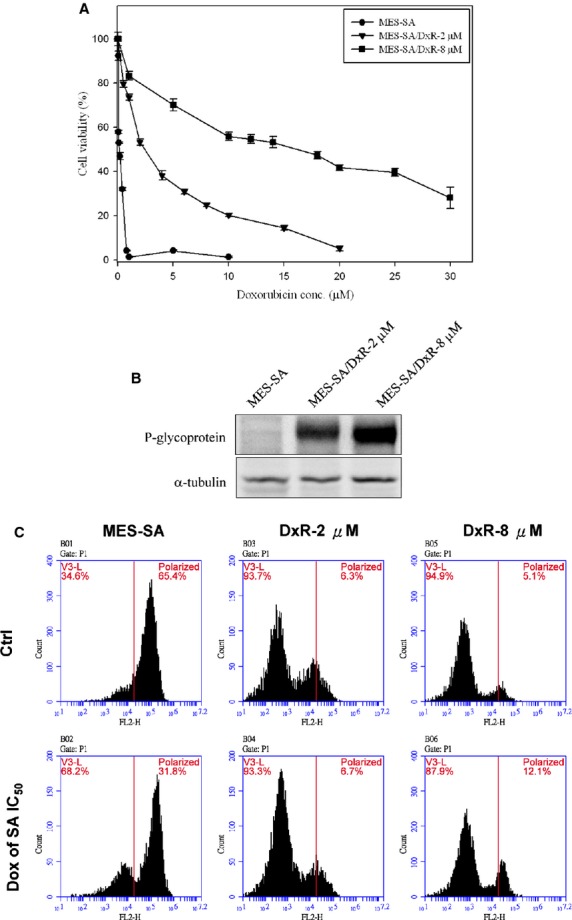
Dose-dependent kinetics of doxorubicin-induced loss of cell viability, increased expression of P-glycoprotein and loss of mitochondrial membrane potential in MES-SA, MES-SA/Dx-2 μM and MES-SA/Dx-8 μM cells. (A) MES-SA, MES-SA/Dx-2 μM and MES-SA/Dx-8 μM cells grown overnight were treated with a range of doses of doxorubicin and cell viability was determined by MTT assay. The error bars mean SD and were calculated based on 4 independent measurements. (B) Expression of P-glycoprotein in MES-SA, MES-SA/Dx-2 μM and MES-SA/Dx-8 μM cells were monitored by immunoblotting. (C) MES-SA, MES-SA/Dx-2 μM and MES-SA/Dx-8 μM cells were treated with/without 0.25 μM doxorubicin and mitochondrial membrane potentials of these cells were determined by fluorescence-based JC-10 assay.

For this study, we prepared a doxorubicin-sensitive uterine cancer cell line, MES-SA, by maintaining in a doxorubicin-free medium containing 10% (v/v) foetal bovine serum. The MES-SA-resistant cell lines, MES-SA/Dx-2 μM and MES-SA/Dx-8 μM, were grown under continuous exposure to 0.2 and 0.8 μM doxorubicin, respectively, to maintain the multiple drug-resistance phenotypes, and the cells were maintained in a drug-free medium for at least 2 weeks prior to perform experiments in this study. The IC_50_ of MES-SA, MES-SA/Dx-2 μM and MES-SA/Dx-8 μM cells were 0.25 μM, 5.31 μM and 18.75 μM respectively (Fig.1A). MES-SA/Dx-2 μM and MES-SA/Dx-8 μM cells showed a significant up-regulation in P-glycoprotein levels (Fig.1B), demonstrating a difference in doxorubicin resistance across the 3 groups of cells. Followed experiment based on fluorescence mitochondrial potential assay demonstrated that MES-SA significantly loss its mitochondrial membrane potential in comparison with MES-SA/Dx-2 μM and MES-SA/Dx-8 μM cells under treatment of doxorubicin (Fig.1C). These distinctly different physiological and biochemical characteristics made these cell lines suitable for chemotherapy-resistant cell model for a doxorubicin-resistance-associated research.

### 2D-DIGE and MALDI-TOF MS analysis of the mitochondrial proteomes across MES-SA, MES-SA/Dx-2 μM and MES-SA/Dx-8 μM cells

**Fig 2:**
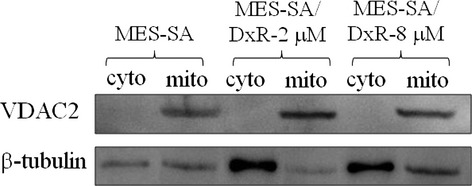
Analysis of purity of the mitochondrial protein extracts by immunoblotting analysis. Mitochondrial fractions were prepared from MES-SA, MES-SA/Dx-2 μM and MES-SA/Dx-8 μM cells. Purity of mitochondrial fractions was determined by immunoblotting analysis by using antibodies against cytoplasmic localized marker protein: beta-tubulin and a mitochondrial localized marker protein: VDAC 2.

For mitochondrial proteome analysis, MES-SA, MES-SA/Dx-2 μM and MES-SA/Dx-8 μM were grown on cell culture dishes and the confluency of cells was checked prior to perform mitochondrial protein extraction. To examine the efficiency of mitochondrial protein extraction, immunoblotting analysis of the beta-tubulin (cytosolic marker) and VDAC2 (mitochondrial marker) levels were carried out and the results showed the mitochondrial fractions were significantly enriched (Fig.2). The 2D-DIGE analysis shows that there is a significant protein expression profiling changes between the mitochondrial fraction and cytosolic fraction indicating the established mitochondrial enrichment is suitable for providing downstream mitochondrial proteome analysis (data not shown).

**Fig 3:**
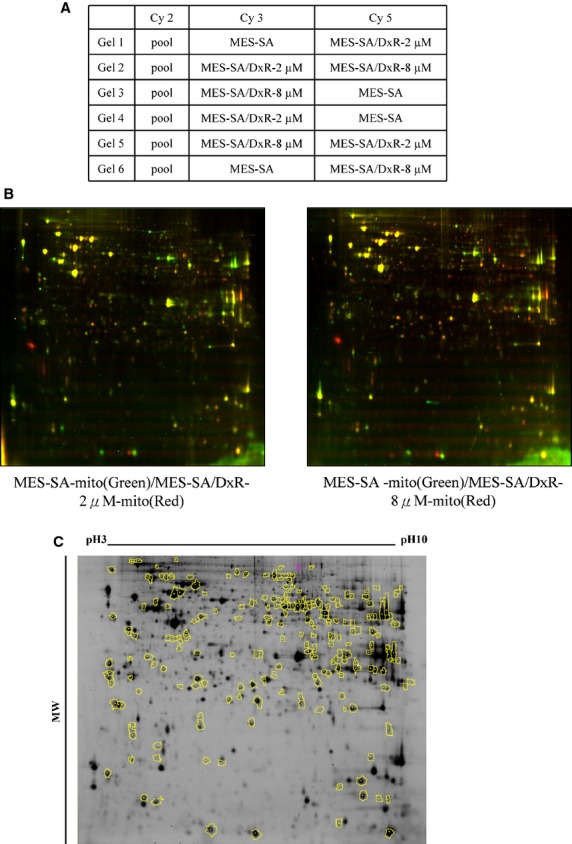
Analysis of the uterine mitochondrial proteomes of MES-SA, MES-SA/Dx-2 μM and MES-SA/Dx-8 μM cells by 2D-DIGE. Mitochondrial proteins (150 μg each) purified from MES-SA, MES-SA/Dx-2 μM and MES-SA/Dx-8 μM cells were labelled with Cy-dyes and separated by using 24 cm, pH 3–10 non-linear IPG strips followed by resolved with 12.5% SDS-PAGE. (A) Samples arrangement for a triplicate 2D-DIGE experiment. (B) 2D-DIGE images of MES-SA, MES-SA/Dx-2 μM and MES-SA/Dx-8 μM at appropriate excitation and emission wavelengths were pseudo-coloured and overlaid with ImageQuant Tool (GE Healthcare). (C) The differentially expressed protein features are annotated with yellow circles.

**Fig 4:**
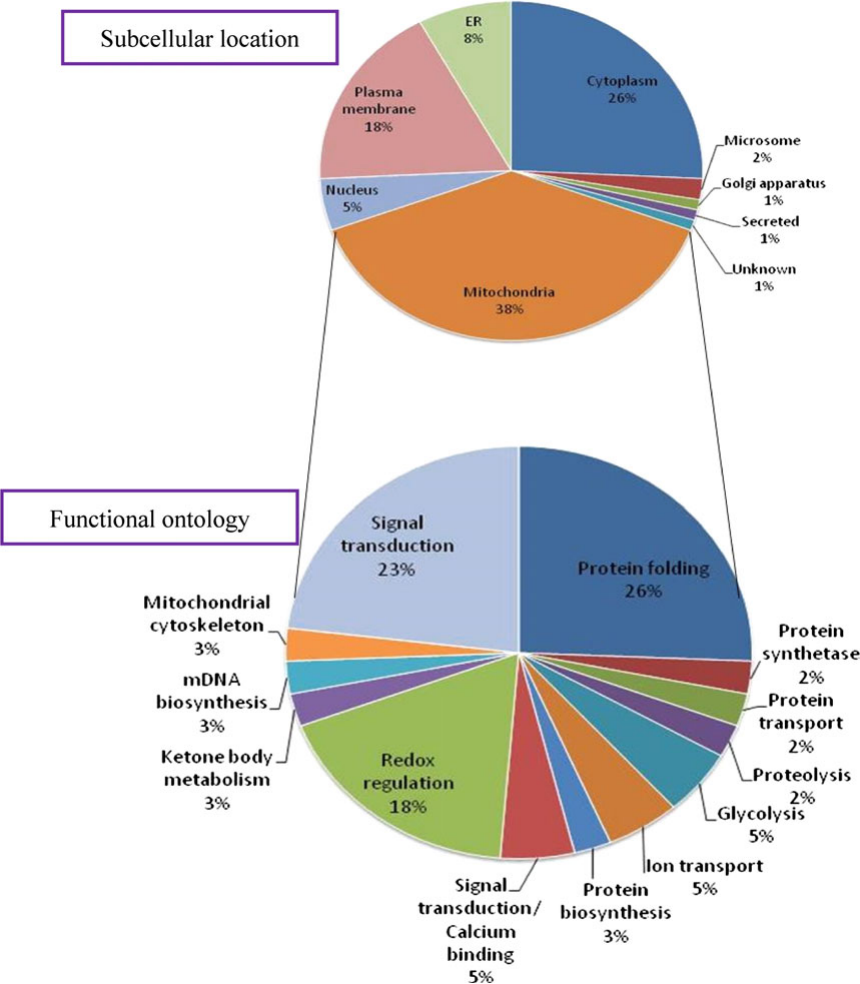
Percentage of intracellular locations of isolated mitochondrial fractions identified by 2D-DIGE/MALDI-TOF MS for MES-SA, MES-SA/Dx-2 μM and MES-SA/Dx-8 μM cells (A). Percentage of identified mitochondrial proteins according to their biological functions (B).

To identify altered abundance of mitochondrial proteins and relate it to the formation of chemotherapy drug resistance-induced by doxorubicin, the mitochondrial proteomic profiles of MES-SA, MES-SA/Dx-2 μM and MES-SA/Dx-8 μM were analysed. The triplicates of the three different mitochondrial fractions from MES-SA, MES-SA/Dx-2 μM and MES-SA/Dx-8 μM cells were compared by 2D-DIGE to have a comprehensive overview of uterine cell drug resistance. After image analysis with DeCyder v7.0, more than 1704 protein spots were well-defined (Fig.3). To reduce the intrinsic variability derived from protein samples and gel-to-gel variation, only those protein features appeared at least in all of the triplicate gel images were selected for statistical analysis. Furthermore, biological variation analysis of spots showing greater than 1.5-fold change in expression with a *t*-test score of less than 0.05 were visually checked before confirming the alterations for protein identification. MALDI-TOF MS identification revealed 101 unique differentially expressed proteins across MES-SA, MES-SA/Dx-2 μM and MES-SA/Dx-8 μM (data not shown). In which, 39 (39%) identified proteins were dominantly mitochondria-located proteins. Of the 39 mitochondrial proteins, 25 of these proteins were shown doxorubicin dose-dependent changes across MES-SA, MES-SA/Dx-2 μM and MES-SA/Dx-8 μM (Fig.4 and Table1). In these mitochondrial proteins, most of them were involved in protein folding, signal transduction and redox regulation (Fig.4). Importantly, a number of identified spots such as serine hydroxymethyltransferase and malate dehydrogenase have not been reported in drug-resistance associated study in our knowledge means these proteins are putative mitochondrial regulatory proteins in response to the development of drug resistance. As expect, some of well known resistance markers such as fumarate hydratase were also identified in this 2D-DIGE experiment 12.

**Table 1:** Alphabetical list of identified differentially expressed mitochondrial proteins between doxorubicin-sensitive uterine cancer cells (MES-SA) and doxorubicin-resistant uterine cancer cells (MES-SA/Dx-2 μM and MES-SA/Dx-8 μM) obtained after 2D-DIGE coupled with MALDI-TOF mass spectrometry analysis

Spot no.	Swiss-Prot no.	Gene name	Protein name	pI	MW	No. match. peptides/supplied peptides	Cov. (%)	Score	Functional ontology	MES-SA 2 μM/MES-SA*	*T*-test	MES-SA 8 μM/MES-SA*	T-test	Represen-tative matched peptides†
1602	P61604	HSPE1	10 kD heat shock protein, mitochondrial/Hsp10/CPN10	8.89	10925	8/38	52%	109/56	Stress response	1.76	0.000014	1.74	0.00017	VLLPEYGGTK/VVLDDKDYFLFR
1281	Q99714	HSD17B10	3-hydroxyacyl-CoA dehydrogenase type-2/HADH2/MRPP2	7.66	27134	9/34	37%	82/56	tRNA maturation	1.42	0.048	1.68	0.015	GQTHTLEDFQR/VCNFLASQVPFPSR
430	P10809	HSPD1	60 kD heat shock protein, mitochondrial/HSP60	5.7	61187	6/17	11%	68/56	Protein folding	1.56	0.000011	1.63	2.1E-06	APGFGDNR/GANPVEIR
1669	P10809	HSPD1	60 kD heat shock protein, mitochondrial/HSP60	5.7	61187	9/37	22%	108/56	Protein folding	1.77	0.00000028	1.83	1.3E-06	LVQDVANNTNEEAGDGTTT/GYISPYFINTSK
844	P24752	ACAT1	Acetyl-CoA acetyltransferase, mitochondrial/Acetoacetyl-CoA thiolase/ACAT	8.98	45456	6/17	18%	81/56	Ketone body metabolism	2.27	0.00000084	2.38	1.1E-06	LNVTPLAR/GSTPYGGVK
884	O15382	BCAT2	Branched-chain-amino-acid aminotransferase, mitochondrial/BCATM	8.88	44658	7/25	22%	71/56	Amino acids catabolism	1.37	0.0031	1.73	0.0011	EIQYGIR/LELLECIR
1695	O75390	CS	Citrate synthase, mitochondrial	8.45	51908	4/18	9%	57/56	TCA cycle	1.72	0.00012	1.63	0.00012	AYAQGISR/ALGFPLERPK
1241	Q9NX63	CHCHD3	Coiled-coil-helix-coiled-coil-helix domain-containing protein 3,mitochondrial	8.48	26421	8/22	34%	85/56	Mitochondrial cytoskeleton	1.89	0.00012	1.85	0.0025	YSGAYGASVSDEELKR/ILQCYR
703	P31930	UQCRC1	Cytochrome b-c1 complex subunit 1, mitochondrial/Complex III subunit 1	5.94	53297	10/23	21%	92/56	Electron transport	1.68	0.00000088	1.8	3.3E-06	IPLAEWESR/RIPLAEWESR
791	P22695	UQCRC2	Cytochrome b-c1 complex subunit 2, mitochondrial/Complex III subunit 2	8.74	48584	7/15	20%	98/56	Electron transport	1.79	0.0012	1.93	0.0011	AVAFQNPQTHVIENLHAAAYR/VTSEELHYFVQNHFTSAR
1310	P47985	UQCRFS1	Cytochrome b-c1 complex subunit Rieske, mitochondrial	8.55	29934	6/31	16%	62/56	Electron transport	1.61	0.000021	1.55	0.00072	VPDFSEYR/VPDFSEYRR
1700	P49411	TUFM	Elongation factor Tu, mitochondrial/EF-Tu/P43	7.26	49852	11/45	32%	128/56	Protein biosynthesis	1.59	0.00000029	1.66	5.2E-07	AEAGDNLGALVR/GTVVTGTLER
1238	P30084	ECHS1	Enoyl-CoA hydratase, mitochondrial/Short-chain enoyl-CoA hydratase/SCEH	8.34	31823	7/20	23%	79/56	Fatty acid metabolism	1.44	0.00002	1.5	0.000038	AQFAQPEILIGTIPGAGGTQR/LFYSTFATDDRK
779	P07954	FH	Fumarate hydratase, mitochondrial/Fumarase	8.85	54773	8/19	17%	81/56	TCA cycle	1.9	0.00000051	2.18	2.5E-07	YYGAQTVR/IEYDTFGELK
1132	Q16836	HADH	Hydroxyacyl-coenzyme A dehydrogenase, mitochondrial/HADHSC	8.88	34329	6/15	14%	62/56	Fatty acid metabolism	1.3	0.0043	1.53	0.00019	GIEESLR/DTPGFIVNR
991	P50213	IDH3A	Isocitrate dehydrogenase [NAD] subunit alpha, mitochondrial/Isocitric dehydrogenase subunit alpha	6.47	40022	7/33	17%	78/56	TCA cycle	−1.58	0.024	−1.3	0.028	CSDFTEEICR/IAEFAFEYAR
993	P40926	MDH2	Malate dehydrogenase, mitochondrial	8.92	35937	10/35	34%	106/56	TCA cycle	2.65	0.00000013	2.9	1.2E-07	EGVVECSFVK/LSALARPASAALR
1003	P40926	MDH2	Malate dehydrogenase, mitochondrial	8.92	35937	8/14	26%	93/56	TCA cycle	1.53	0.012	1.56	0.024	GYLGPEQLPDCLK/AGAGSATLSMAYAGAR
1006	P40926	MDH2	Malate dehydrogenase, mitochondrial	8.92	35937	6/24	20%	68/56	TCA cycle	1.82	0.0052	1.89	0.003	MLSALARPASAALR/IFGVTTLDIVR
1043	P40926	MDH2	Malate dehydrogenase, mitochondrial	8.92	35937	5/18	19%	72/56	TCA cycle	1.87	0.00014	1.9	0.00026	GYLGPEQLPDCLK/VDFPQDQLTALTGR
1263	O75431	MTX2	Metaxin-2/Mitochondrial outer membrane import complex protein 2	5.9	30086	6/18	14%	70/56	Protein transport	1.65	0.0004	1.52	0.0005	QWEVKR/IEQHYFEDR
200	Q7Z3X1	IMMT	Mitochondrial inner membrane protein/HMP	6.08	84025	10/29	12%	100/56	Calcium homoeostasis	1.44	0.000094	1.52	0.00034	QAAAHTDHLR/WDSHFR
218	Q7Z3X1	IMMT	Mitochondrial inner membrane protein/HMP	6.08	84025	11/38	13%	69/56	Calcium homoeostasis	1.66	0.00025	1.63	0.00013	FVNQLKGESR/GVYSEETLR
607	Q10713	PMPCA	Mitochondrial-processing peptidase subunit alpha/Alpha-MPP/MPPA	6.45	58729	15/43	28%	131/56	Proteolysis	1.43	0.047	1.77	0.0046	LAFSSTAR/HGGICDCQTSR
1236	O75489	NDUFS3	NADH dehydrogenase [ubiquinone] iron-sulphur protein 3, mitochondrial/NADH-ubiquinone oxidoreductase 30 kD subunit	6.99	30337	7/46	27%	76/56	Electron transport	2.05	0.00017	1.98	0.00023	AANWYER/ESAGADTRPTVRPR
260	P28331	NDUFS1	NADH-ubiquinone oxidoreductase 75 kD subunit, mitochondrial/CI-75kD	5.89	80443	21/41	25%	158/56	Electron transport	1.77	0.000033	1.95	0.0001	FEAPLFNAR/FCYHER
786	P04181	OAT	Ornithine aminotransferase, mitochondrial/Ornithine delta-aminotransferase	6.57	48846	14/41	35%	148/56	Amino acid biosynthesis	1.53	0.026	1.78	0.0057	IVFAAGNFWGR/FAPPLVIK
1212	P35232	PHB	Prohibitin	5.57	29843	10/29	35%	123/56	DNA synthesis inhibition	1.83	0.00000054	1.73	1.1E-06	AAELIANSLATAGDGLIELR/FDAGELITQR
1129	P32322	PYCR1	Pyrroline-5-carboxylate reductase 1, mitochondrial/P5CR 1	7.18	33568	7/28	26%	87/56	Amino acids biosynthesis	1.68	0.00015	1.92	0.000035	DNVSSPGGATIHALHVLESGGFR/SLLINAVEASCIR
820	Q9UBU0	PDHA1	Pyruvate dehydrogenase E1 component subunit alpha, somatic form,mitochondrial/PHE1A	8.35	43952	10/29	22%	97/56	Glycolysis	1.25	0.013	1.91	0.000086	RGDFIPGLR/FAAAYCR
559	P34897	SHMT2	Serine hydroxymethyl-transferase, mitochondrial/SHMT	8.76	56414	18/30	25%	162/56	mDNA biosynthesis	1.58	0.000053	1.51	0.00064	MREVCDEVK/QRVEQFAR
852	Q9UJZ1	STOML2	Stomatin-like protein 2/SLP-2	6.88	38624	5/17	17%	70/56	Unknown	1.95	0.00000003	1.75	7.9E-06	ATVLESEGTR/DIHVPPR
867	Q9UJZ1	STOML2	Stomatin-like protein 2/SLP-2	6.88	38624	5/22	18%	61/56	Unknown	1.91	0.0000015	1.78	4.8E-06	ILEPGLNILIPVLDR/NTVVLFVPQQEAWVVER
334	Q9UC56	HSPA9	Stress-70 protein, mitochondrial/HSPA9B/Mortalin	5.87	73920	14/29	23%	117/56	Protein folding	1.44	0.0000029	1.52	5.3E-06	YAEEDRR/DNMALQR
370	P31040	SDHA	Succinate dehydrogenase [ubiquinone] flavoprotein subunit, mitochondrial/SDHF	7.06	73672	19/57	33%	154/56	Electron transport	2.01	2.6E-08	2.19	3.1E-07	TGHSLLHTLYGR/DHVYLQLHHLPPEQLATR
1227	P21912	SDHB	Succinate dehydrogenase [ubiquinone] iron-sulphur subunit, mitochondrial/Iron-sulphur subunit of complex II/Ip	9.03	32407	7/24	15%	62/56	Electron transport	2.26	0.0069	2.08	0.000015	WMIDSR/DDFTEER
1301	P30048	PRDX3	Thioredoxin-dependent peroxide reductase, mitochondrial/Peroxiredoxin-3/AOP1	7.67	28017	4/25	17%	63/56	Redox regulation	1.38	0.00094	1.59	0.000028	SVEETLR/DYGVLLEGSGLALR
1302	P30048	PRDX3	Thioredoxin-dependent peroxide reductase, mitochondrial/Peroxiredoxin-3/AOP1	7.67	28017	4/15	17%	62/56	Redox regulation	−1.76	0.000022	−1.44	0.00053	DYGVLLEGSGLALR/HLSVNDLPVGR
1133	P21796	VDAC1	Voltage-dependent anion-selective channel protein/Outer mitochondrial membrane protein porin 1	8.62	30868	12/46	46%	121/56	Ion transport	1.29	0.00079	1.53	0.000039	VTGSLETKYR/LTLSALLDGKNVNAGGHK

* Average ratio of differential expression (≧1.5-fold increase or decrease) between doxorubicin-resistant uterine cancer cells (MES-SA/Dx-2 μM or MES-SA/Dx-8 μM) and doxorubicin-sensitive uterine cancer cells (MES-SA). Grey shaded cells indicate proteins where the changes between MES-SA/Dx-8 μM and MES-SA are significantly greater than the changes between MES-SA/Dx-2 μM and MES-SA.

† In MS analysis, we listed 2 representative peptide sequences in the matched peptide column. Student's *T*-test has been used in the data analysis.

### Validation of characterized resistance-associated mitochondrial proteins *via* immunoblotting

**Fig 5:**
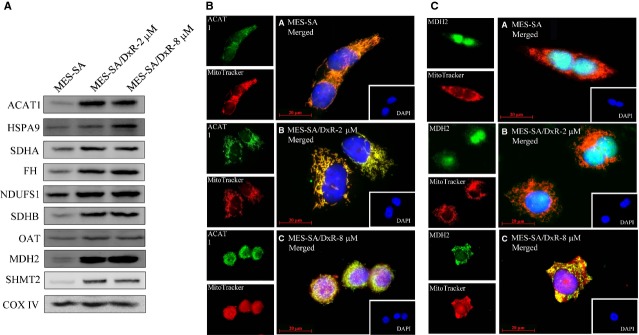
Representative immunoblotting and immunofluorescence analyses for selected differentially expressed proteins identified by proteomic analysis in MES-SA, MES-SA/Dx-2 μM and MES-SA/Dx-8 μM cells. (A) The levels of identified proteins including ACAT1, HSPA9, SDHA, FH, NDUFS1, SDHB, OAT, MDH2 and SHMT2 were validated by immunoblotting while COX IV was used as an internal control in the validation. (B) 5 × 10^4^ MES-SA, MES-SA/Dx-2 μM and MES-SA/Dx-8 μM cells were seed on cover slips before fixation and staining for DAPI, mitotracker and ACAT1. Each set of fields were taken by using the same exposure and images are representative of 6 different fields; scale bar = 20 μm. (C) 5 × 10^4^ MES-SA, MES-SA/Dx-2 μM and MES-SA/Dx-8 μM cells were seed on cover slips before fixation and staining for DAPI, mitotracker and MDH2. Each set of fields were taken by using the same exposure and images are representative of 6 different fields; scale bar = 20 μm.

The proteomic study identified a number of doxorubicin resistance-associated mitochondrial proteins. It was essential to confirm the expression of these proteins by using independent biochemical experiments. To this end, we performed immunoblotting analyses to confirm the expression levels of acetyl-CoA acetyltransferase (ACAT1), stress-70 protein (HSPA9), succinate dehydrogenase [ubiquinone] flavoprotein subunit (SDHA), fumarate hydratase (FH), NADH-ubiquinone oxidoreductase 75 kD subunit (NDUFS1), succinate dehydrogenase [ubiquinone] iron-sulphur subunit (SDHB), ornithine aminotransferase (OAT), malate dehydrogenase (MDH2) and serine hydroxymethyltransferase (SHMT2) obtained from the mitochondrial fractions of MES-SA, MES-SA/Dx-2 μM and MES-SA/Dx-8 μM (Fig.5A). All of these proteins were up-regulated in the mitochondrial fractions of MES-SA/Dx-2 μM and MES-SA/Dx-8 μM, compared to the expression levels present in MES-SA. These results confirmed the differential resistance across MES-SA, MES-SA/Dx-2 μM and MES-SA/Dx-8 μM. In general, our immunoblotting results are in agreement with the proteomic analysis results.

To further verify the protein expression levels of unreported identified proteins across MES-SA, MES-SA/Dx-2 μM and MES-SA/Dx-8 μM, the expression of mitochondrial protein ACAT1 was examined and the immunofluorescence result demonstrated that ACAT1 was located in mitochondria due to the spatial overlapping of ACAT1 with mitotracker and its expression is up-regulated in MES-SA/Dx-2 μM and MES-SA/Dx-8 μM than in MES-SA implying ACAT1 is over-expressed in doxorubicin-induced drug resistant uterine cancer cells (Fig.5B). Thus, ACAT1 is potentially to be a candidate for the diagnosis of doxorubicin-associated drug resistance in uterine cancer. Notably, ACAT1, a major mitochondria-located protein, was dominantly distributed in the mitochondria of MES-SA, MES-SA/Dx-2 μM and MES-SA/Dx-8 μM cells and was gradually increasing its expression level in response to the drug resistance with a positive correlation (Fig.5B).

Further investigation demonstrated that the level of mitochondria-located mitochondrial protein MDH2 was significantly increased in resistant line MES-SA/Dx-8 μM rather than in MES-SA or in MES-SA/Dx-2 μM implying MDH2 is over-expressed in highly resistant uterine cancer cells (Fig.5C). Thus, MDH2 is potentially to be a mitochondrial marker for the diagnosis of doxorubicin-associated drug resistance in uterine cancer. Importantly, MDH2 was dominantly distributed in the nucleus in both MES-SA and MES-SA/Dx-2 μM cells, but was gradually shifting to mitochondria in MES-SA/Dx-8 μM cells (Fig.5C) implying the intracellular distribution of MDH2 is associated with doxorubicin-induced drug resistance.

### Evaluation of the roles of ACAT1 and MDH2 on doxorubicin resistance in uterine cancer using siRNA knockdown

**Fig 6:**
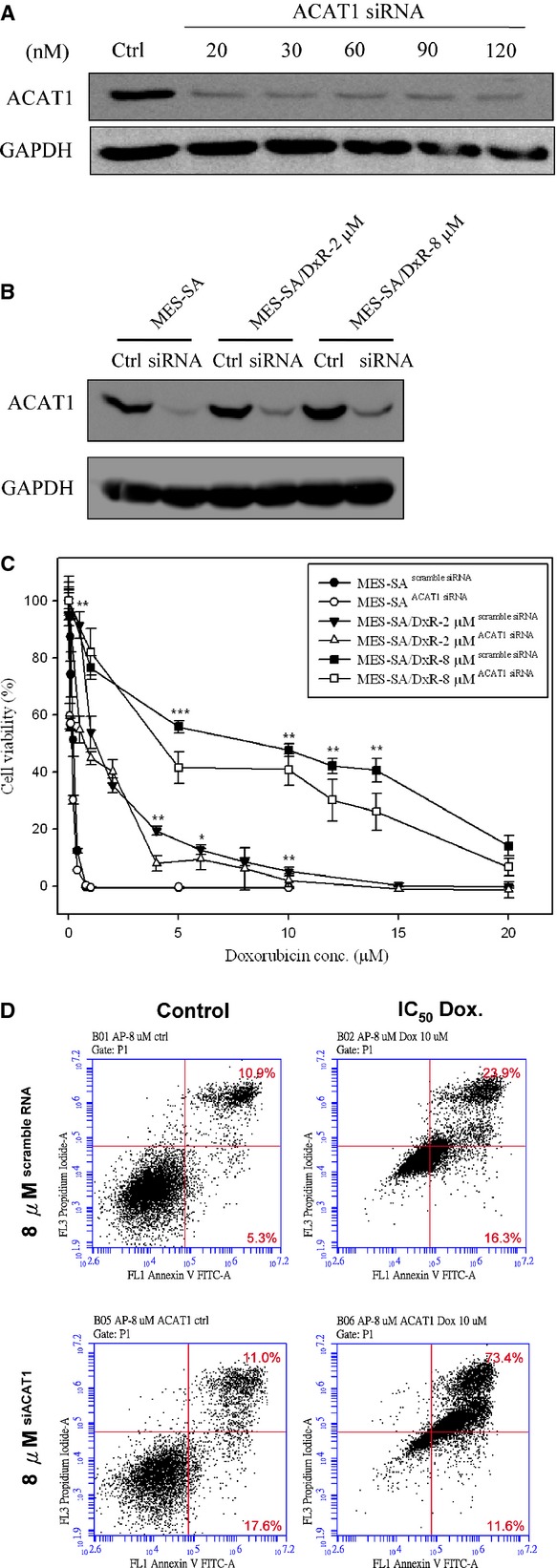
Effect of doxorubicin on cell viability of ACAT1 siRNA-silenced MES-SA, MES-SA/Dx-2 μM and MES-SA/Dx-8 μM cells. (A) Efficiency of ACAT1 siRNA on the inhibition of ACAT1 expression in MES-SA/Dx-8 μM cells. MES-SA/Dx-8 μM cells grown overnight were treated with indicated concentrations of ACAT1-specific siRNA for 24 hrs. Expression of ACAT1 in MES-SA/Dx-8 μM cells were monitored with immunoblotting by using primary antibodies against ACAT1. (B) MES-SA, MES-SA/Dx-2 μM and MES-SA/Dx-8 μM cells grown overnight were pre-treated with 20 nM ACAT1-specific siRNA or scramble siRNA with similar GC content. Expression of ACAT1 in MES-SA, MES-SA/Dx-2 μM and MES-SA/Dx-8 μM cells were monitored by immunoblotting by using primary antibodies against ACAT1. (C) MTT-based viability assays were performed where 5000 MES-SA, MES-SA/Dx-2 μM and MES-SA/Dx-8 μM cells seeded into 96-well plate for overnight incubation followed by pre-treated with 20 nM ACAT1-specific siRNA combining with corresponding scramble siRNA. After 24 hrs, cells were treated with indicated concentrations of doxorubicin for 24 hrs followed by incubated with MTT and then DMSO added and the plates shaken for 20 min. followed by measurement of the absorbance at 540 nm. Values were normalized against the untreated samples and are the average of 4 independent measurements ± SD. (D) MES-SA/Dx-8 μM cells were treated with IC_50_ concentrations of doxorubicin or left untreated for 48 hrs. After treatment, 10^6^ cells were incubated with Alexa Fluor 488 and propidium iodide (PI) in 1× binding buffer at room temperature for 15 min., and then stained cells were analysed by flow cytometry to examine effect of doxorubicin on apoptosis in MES-SA/Dx-8 μM cells and ACAT1 siRNA silenced MES-SA/Dx-8 μM cells. Annexin V is presented in *x*-axis as FL1-H, and PI is presented in *y*-axis as FL2-H. LR quadrant indicates the percentage of early apoptotic cells (Annexin V positive cells), and UR quadrant indicates the percentage of late apoptotic cells (Annexin V positive and PI positive cells).

**Fig 7:**
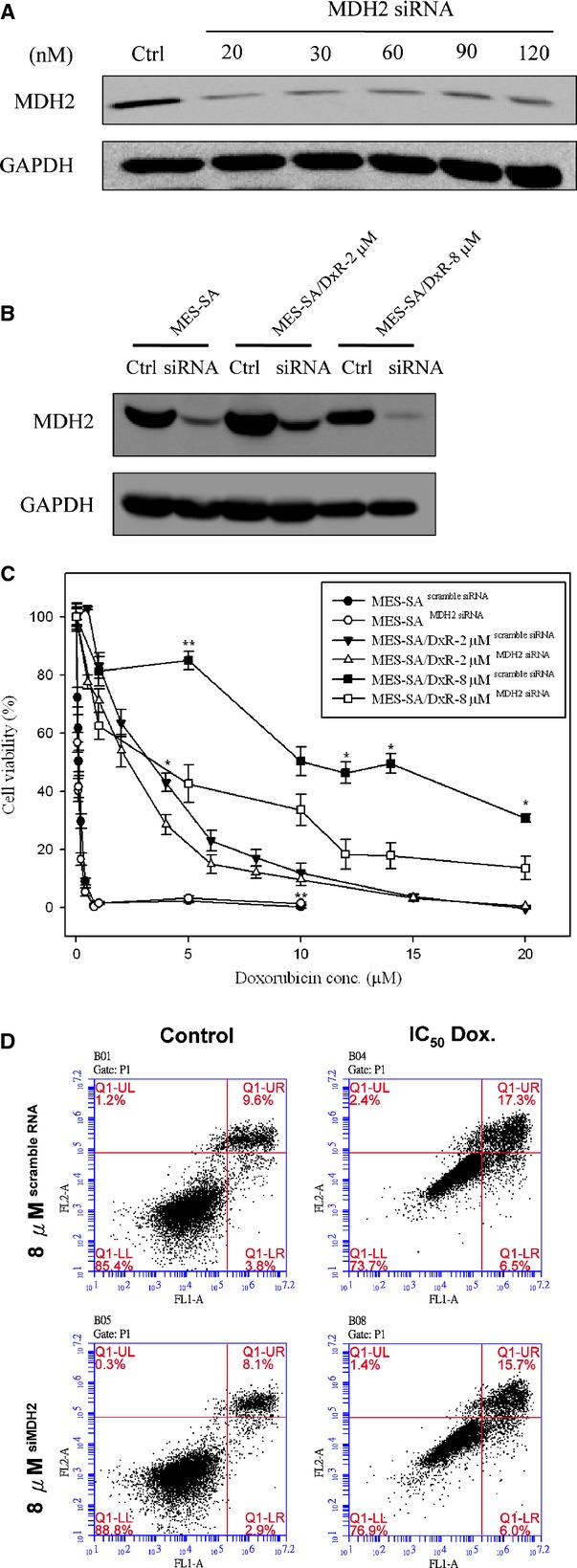
Effect of doxorubicin on cell viability of MDH2 siRNA-silenced MES-SA, MES-SA/Dx-2 μM and MES-SA/Dx-8 μM cells. (A) Efficiency of MDH2 siRNA on the inhibition of MDH2 expression in MES-SA/Dx-8 μM cells. MES-SA/Dx-8 μM cells grown overnight were treated with indicated concentrations of MDH2-specific siRNA for 24 hrs. Expression of MDH2 in MES-SA/Dx-8 μM cells were monitored with immunoblotting by using primary antibodies against MDH2. (B) MES-SA, MES-SA/Dx-2 μM and MES-SA/Dx-8 μM cells grown overnight were pre-treated with 20 nM MDH2-specific siRNA or scramble siRNA with similar GC content. Expression of MDH2 in MES-SA, MES-SA/Dx-2 μM and MES-SA/Dx-8 μM cells were monitored by immunoblotting by using primary antibodies against MDH2. (C) MTT-based viability assays were performed where 5000 MES-SA, MES-SA/Dx-2 μM and MES-SA/Dx-8 μM cells seeded into 96-well plate for overnight incubation followed by pre-treated with 20 nM MDH2-specific siRNA combining with corresponding scramble siRNA. After 24 hrs, cells were treated indicated concentrations of doxorubicin for 24 hrs followed by incubated with MTT and then DMSO added and the plates shaken for 20 min. followed by measurement of the absorbance at 540 nm. Values were normalized against the untreated samples and are the average of 4 independent measurements ± SD. (D) MES-SA/Dx-8 μM cells were treated with IC_50_ concentrations of doxorubicin or left untreated for 48 hrs. After treatment, 10^6^ cells were incubated with Alexa Fluor 488 and propidium iodide (PI) in 1× binding buffer at room temperature for 15 min., and then stained cells were analysed by flow cytometry to examine effect of doxorubicin on apoptosis in MES-SA/Dx-8 μM cells and MDH2 siRNA silenced MES-SA/Dx-8 μM cells. Annexin V is presented in *x*-axis as FL1-H, and PI is presented in *y*-axis as FL2-H. LR quadrant indicates the percentage of early apoptotic cells (Annexin V positive cells), and UR quadrant indicates the percentage of late apoptotic cells (Annexin V positive and PI positive cells).

We found that mitochondrial proteins, ACAT1 and MDH2 are over-expressed in the mitochondrial fractions of the doxorubicin-resistant cells compared to doxorubicin-sensitive MES-SA cells; these 2 proteins are among the most differentially expressed proteins identified in our proteomic analysis. We performed knockdown experiments in MES-SA, MES-SA/Dx-2 μM and MES-SA/Dx-8 μM cells to evaluate the roles of ACAT1 and MDH2 in doxorubicin resistance. Immunoblotting analysis showed greater than 70% efficiency in the reduction of endogenous ACAT1 and MDH2 protein levels when GAPDH were the internal standards (Figs6A, B and 7A, B). Knockdown of ACAT1 and MDH2 with 20 nM of ACAT1 siRNA and 20 nM of MDH2 siRNA, respectively, or the corresponding control (pGCsi-control) resulted in significantly reduced viability in the MES-SA/Dx-2 μM and MES-SA/Dx-8 μM cells following treatment with the indicated concentrations of doxorubicin compared to scramble siRNA transfected controls (Figs6C and 7C). MTT assay also revealed no significantly decreased viability in ACAT1 and MDH2 knockdown MES-SA cells (Figs6C and7C).

Furthermore, we used flow cytometry with PI staining and annexin V-conjugated Alexa Fluor 488 to analyse the percentages of apoptotic MES-SA/Dx-8 μM cells induced by various concentrations of doxorubicin treatment, with or without ACAT1 or MDH2 knockdown. The total number of apoptotic cells is represented by the numbers of early apoptotic cells plotted in the LR quadrant and late apoptotic cells displayed in the UR quadrant of the resulting histograms. We found that treatment of doxorubicin at the IC_50_ doses increases the percentages of total apoptotic cells (LR + UR) in MES-SA/Dx-8 μM cells from 16.2% to 40.2% compared with changes from 28.6% to 85% in ACAT1 knockdown MES-SA/Dx-8 μM cells. Thus, ACAT1 plays an essential role on the preventing doxorubicin-induced cell apoptosis in doxorubicin-resistant cells (Fig.6D). Further study on MDH2 showed that treatment of MES-SA/Dx-8 μM cells with doxorubicin at the IC_50_ doses increased the numbers of total apoptotic cells (LR + UR) from 13.4% to 23.8%, compared with increases ranging from 11% to 21.7% in MDH2 knockdown MES-SA/Dx-8 μM cells. These results indicated that MDH2 has no direct effect on the preventing doxorubicin-induced cell apoptosis in doxorubicin-resistant cells implying some other mechanisms other than apoptosis are contributed by MDH2 knockdown to regulate cell viability (Fig.7D).

## Discussion

A better understanding of chemotherapy-associated drug-resistance mechanisms is essential for offering opportunities in both drug-resistance detection and therapy. Current understanding demonstrated mitochondria is one of the major targets for chemotherapeutic drugs 14,20. Additionally, mutations within the mitochondrial genomes are reported in most cancers, which are believed to promote chemoresistance 21. So far, most of the functional mechanisms of chemoresistance were supposed from transcription-based researches, rather than from translation-level studies. Thus, in current study, we adopted a mitochondrial proteomic approach based on 2D-DIGE analysis coupled with MALDI-TOF/TOF MS to identify mitochondrial proteins which were differentially expressed in the doxorubicin-resistant human uterine cancer cell lines, MES-SA/Dx-2 μM and MES-SA/Dx-8 μM, and its parental cell line MES-SA, to provide a broad view of protein expression and protein–protein interactions related to doxorubicin resistance in uterine cancer. The identified proteins might serve as potential resistant markers for prognosis or diagnosis of the uterine cancer patients to treatment with doxorubicin or other chemotherapy drugs such as 5-fluorouracil.

In current study, we observed 101 differentially expressed MALDI-TOF/TOF MS identified protein spots from isolated mitochondrial fractions across the 3 cell lines. In which, 39 out of the 101 proteins are mitochondria-located proteins. Although only 39% of the isolated proteins were assigned to be mitochondria located, the percentage is higher than the general total cellular protein analysis (∼5% of total cellular proteins) 17,22,23. Notably, part of cytosolic proteins are able to translocate into mitochondria but are assigned to be cytosolic proteins which might contribute to the low coverage rate of the mitochondrial fraction in total enriched proteins.

This study showed that ACAT1 and MDH2 proteins are potential in the formation of doxorubicin drug resistance. We validated the functions of ACAT1 and MDH2 in drug resistance, and found that their overexpressions contribute significantly to the development of doxorubicin resistance in MES-SA/Dx-2 μM and MES-SA/Dx-8 μM cells. Our studies also demonstrated that multiple mechanisms are correlated with the formation of chemotherapy resistance in uterine cancer, and that these different mechanisms might contribute to chemotherapeutic resistance in the treatment of uterine cancer.

ACAT1 is a mitochondrial enzyme participating in numerous pathways including fatty acid metabolism, synthesis and breakdown of ketone bodies, valine, leucine and isoleucine degradation, lysine degradation, tryptophan metabolism, pyruvate metabolism and butanoate metabolism 24–26. Thus, ACAT1 plays an important role in the modulation of cell physiology. However, there is limited report demonstrated the potency of ACAT1 in either cancer research or chemotherapy resistance. Only one report shows that ACAT1 is a potential diagnostic cancer marker for renal carcinoma 27 with no report in drug resistant field. Our current results indicated that ACAT1 has profound effect in the formation of doxorubicin resistance and further implicated the complication between doxorubicin resistance and these metabolic pathways.

MDH2 is a citric acid cycle enzyme that catalyses the conversion of malate into oxaloacetate as well as involves in gluconeogenesis *via* reducing oxaloacetate into malate and traversing malate across the inner mitochondrial membrane. Additionally, MDH2 is one of the major players in malate-aspartate shuttle which can transfer electrons from cytosolic NADH to produce mitochondrial NADH and generate ATP *via* electron transport chain 28. This implies up-regulation of MDH2 might increase cellular energy to supply biosynthesis of resistance-associated proteins and provide energy for P-glycoprotein to pump-out chemotherapeutic drugs. In addition, Liu *et al*.'s study revealed that MDH2 mediates prostate cancer resistance to docetaxel-chemotherapy through JNK pathway and MDH2 knockdown significantly enhanced the docetaxel sensitivity in prostate cancer cells. Their study also showed that MDH2 knockdown significantly reduced cellular ATP levels and increased the ADP/ATP and NAD^+^/NADH level as well as intracellular ROS concentration 29.

Drug resistant tumour cells have been reported to be more metastatic and invasive as well as increased wound healing ability, when compared with drug sensitive tumour cells. To study the effect of ACAT1 and MDH2 knockdown on wound healing of MES-SA, MES-SA/Dx-2 μM and MES-SA/Dx-8 μM, these cells were knockdown followed by performed wound closure experiments. The results indicated that a significant healing delay was observed in ACAT1 siRNA and MDH2 siRNA treated MES-SA/Dx-2 μM and MES-SA/Dx-8 μM cells. Compared to this condition, the restore of wound areas were found to reduce significantly when MES-SA cells were treated with either ACAT1 siRNA or MDH2 siRNA. These results implied that knockdown of ACAT1 and MDH2 has profound effect on doxorubicin resistance-associated wound healing (Fig. S1).

Mitochondrial hsp70 (HSPA9) is a crucial component in the import and folding of mitochondrial proteins and help to protect cells from stress including chemotherapeutic agents. In comparison with normal cells, most cancer cells overexpress mitochondrial hsp70 (HSPA9) at the basal level to resist to various damages at different stages of tumourigenesis and during anti-cancer treatment 30. This is the reason why mitochondrial hsp70 (HSPA9) has been widely observed in cancer drug-resistance researches 31 as well as in aggressive cancer studies 32. In current study, as expect mitochondrial hsp70 (HSPA9) is significantly overexpressed in doxorubicin-induced drug resistance and might be further evaluated as a target to diminish drug resistance.

**Fig 8:**
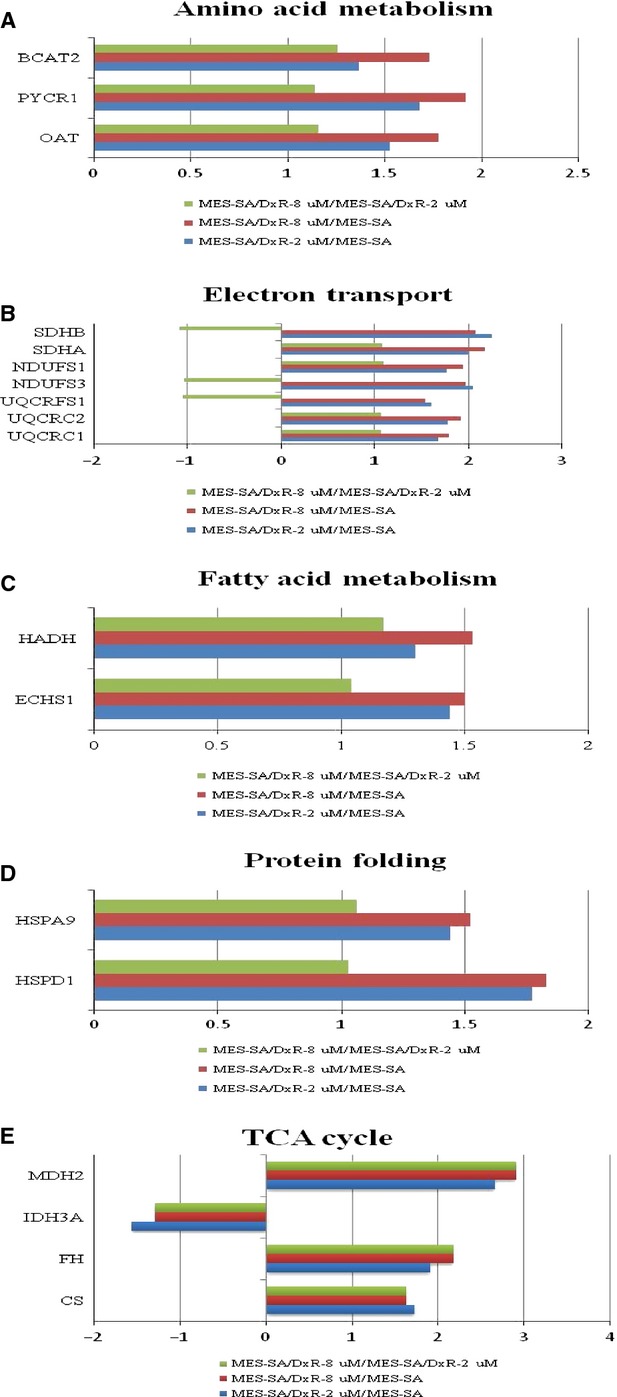
Expression profiles for differentially expressed proteins potentially contributing to: (A) amino acid metabolism, (B) electron transport, (C) fatty acid metabolism, (D) protein folding and (E) TCA cycle in MES-SA, MES-SA/Dx-2 μM and MES-SA/Dx-8 μM. The horizontal bars represent fold-changes in protein expression and the vertical axis indicates the identified proteins. Additional details for each protein can be found in Table1.

In this study, we also determined numerous potential biological functions of the identified proteins towards baseline resistance in human uterine cancer cells by using a Swiss-Prot search combined with KEGG pathway analysis. Our findings should be useful for a systematic study on the mechanisms of doxorubicin resistance in uterine cancer. Figure8 shows a comparison of the expression profiles of the differentially expressed mitochondrial proteins for baseline resistance. Mitochondrial proteins known to regulate amino acid metabolism, electron transport, fatty acid metabolism, protein folding and TCA cycle are up-regulated in resistance cells, but not in sensitive cells, implying that doxorubicin-resistant cells have greater ability to maintain mitochondrial protein conformation, energy metabolism and biomacromolecule metabolism.

Protein species theory is proposed to be the smallest unit of proteome and believed the formation of different forms of proteins (such as post-translational modifications), each of which is able to have specific chemical structure and specific function. The original biosynthetic product of translation stands for a prototype protein species, the initial protein species. This initial protein species is possibly further biochemically processed, modified, or proteolytically and/or secreted out of the cell or transported to intracellular organelles, spliced or degraded. Each of these covalent modifications or processed of a protein leads to a new protein species 33,34. In this study, some of identified proteins including HSP60, MDH2, mitochondrial inner membrane protein, stomatin-like protein 2 and peroxiredoxin-3 have been identified in multiple spots (Table1) implying these proteins encounter differential processes/modifications and form new protein species against initial translational products.

To sum up, our current mitochondrial proteomic approaches allowed us to identify numerous proteins, including ACAT1 and MDH2 involved in various drug-resistance-forming mechanisms. Our results provide useful diagnostic markers and therapeutic candidates for the treatment of doxorubicin-resistant uterine cancer.

## Supporting Information

**Figure S1.** Effect of ACAT1/MDH2 knockdown on wound closure in MES-SA, MES-SA/Dx-2 μM and MES-SA/Dx-8 μM cells.
