# Supplementary figures and images for: Mitochondrial proteomics with siRNA knockdown to reveal ACAT1 and MDH2 in the development of doxorubicin-resistant uterine cancer
Source: J Cell Mol Med. 2015 Jan 30;19(4):744–59. doi: 10.1111/jcmm.12388 (PMC4395189; doi:10.1111/jcmm.12388)

## Slide 1
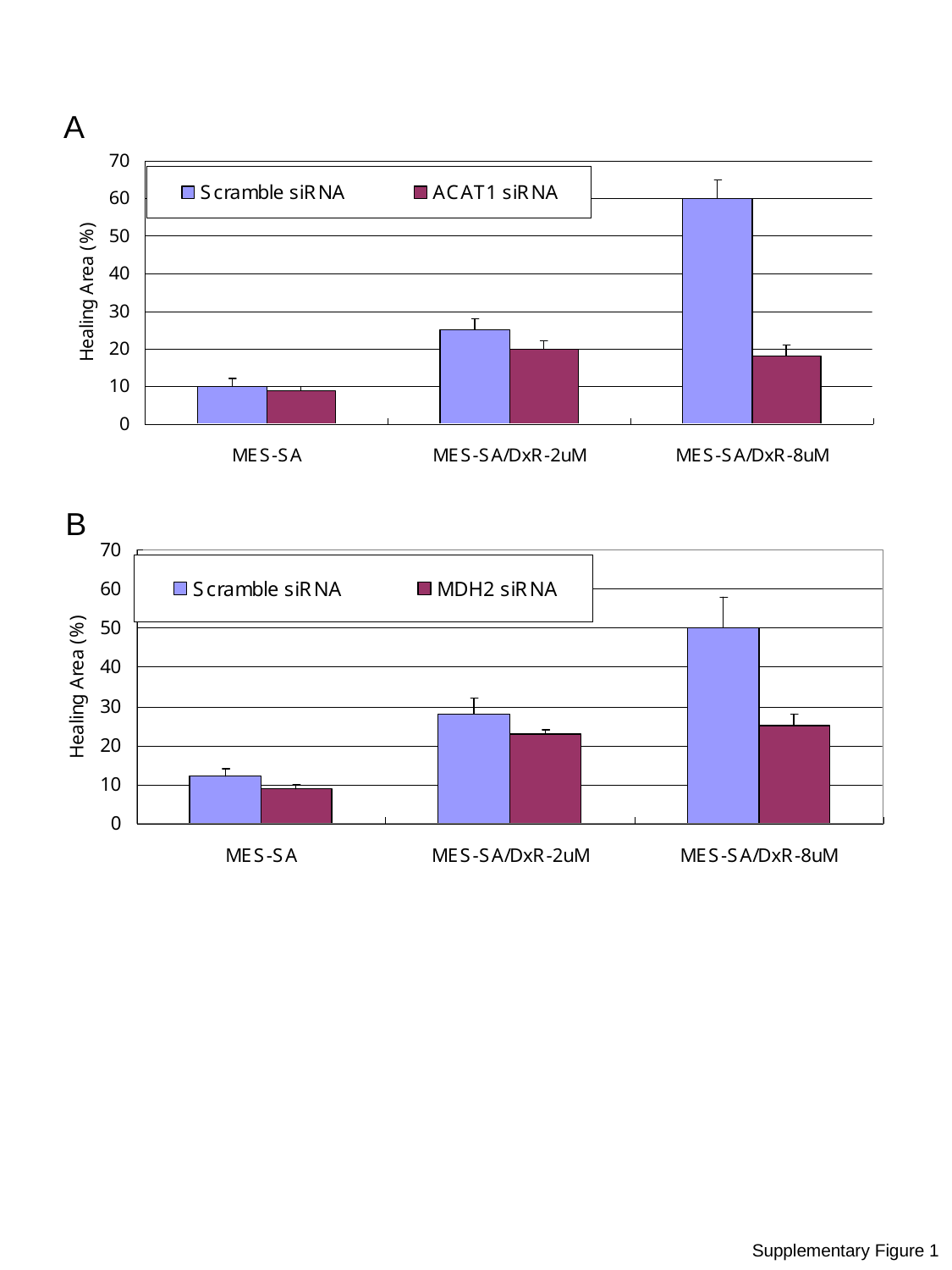

A
B
Supplementary Figure 1

Supplement: Supplementary file 1 [file jcmm0019-0744-sd1.ppt]
